# Clinical and radiological characteristics of acute pulmonary embolus in relation to 28-day and 6-month mortality

**DOI:** 10.1371/journal.pone.0258843

**Published:** 2021-12-28

**Authors:** Lindsey Norton, Gordon Cooper, Owen Sheerins, Killian Mac a’ Bháird, Giles Roditi, Michael Adamson, David Young, Ross Dolan, Colin Church, Adrian Brady, Campbell Tait, Graham McKenzie, Alasdair McFadyen, Matthew Zelic, Donogh Maguire

**Affiliations:** 1 Department of Radiology, NHS Greater Glasgow & Clyde, Glasgow, United Kingdom; 2 Department of Surgery, University Hospital Hairmyres, South Lanarkshire, Scotland, United Kingdom; 3 Department of Orthopaedic Surgery, Glasgow Royal infirmary, Scotland, United Kingdom; 4 Institute of Cardiovascular and Medical Sciences, BHF Glasgow Cardiovascular Research Centre, University of Glasgow, Glasgow, United Kingdom; 5 Emergency Medicine Department, Glasgow Royal Infirmary, Scotland, United Kingdom; 6 Department of Mathematics and Statistics, University of Strathclyde, Glasgow, Scotland, United Kingdom; 7 Academic Unit of Surgery, School of Medicine, University of Glasgow, Glasgow, Scotland, United Kingdom; 8 Department of Respiratory Medicine, Queen Elizabeth University Hospital, Scotland, United Kingdom; 9 Department of Cardiology, Glasgow Royal infirmary, Scotland, United Kingdom; 10 Department of Haematology, Glasgow Royal infirmary, Scotland, United Kingdom; Chang Gung Memorial Hospital and Chang Gung University, Taoyuan, Taiwan, TAIWAN

## Abstract

**Background:**

Patients with acute pulmonary embolism (PE) exhibit a wide spectrum of clinical and laboratory features when presenting to hospital and pathophysiologic mechanisms differentiating low-risk and high-risk PE are poorly understood.

**Objectives:**

To investigate the prognostic value of clinical, laboratory and radiological information that is available within routine tests undertaken for patients with acute PE.

**Methods:**

Electronic patient records (EPR) of patients who underwent Computed Tomography Pulmonary Angiogram (CTPA) scan for the investigation of acute PE during 6-month period (01.01.2016–30.06.2016) were examined. Data was gathered from EPR for patients that met inclusion criteria and all CTPA scans were re-evaluated. Biochemical thresholds of low-grade and high-grade inflammation, serum CRP >10mg/L and >150mg/L and serum albumin concentrations <35g/L and <25 g/L, were combined in the Glasgow Prognostic Score (GPS) and peri-operative Glasgow Prognostic Score (poGPS) respectively. Neutrophil Lymphocyte ratio (NLR) was also calculated. Pulmonary Embolus Severity Index score was calculated.

**Results:**

Of the total CTPA reports (n = 2129) examined, 245 patients were eligible for inclusion. Of these, 20 (8%) patients had died at 28-days and 43 (18%) at 6-months. Of the 197 non-cancer related presentations, 28-day and 6-month mortality were 3% and 8% respectively. Of the 48 cancer related presentations, 28-day and 6-month mortality were 29% and 58% respectively. On univariate analysis, age ≥65 years (p<0.01), PESI score ≥100(p = <0.001), NLR ≥3(p<0.001) and Coronary Artery Calcification (CAC) score ≥ 6 (p<0.001) were associated with higher 28-day and 6-month mortality. PESI score ≥100 (OR 5.2, 95% CI: 1.1, 24.2, P <0.05), poGPS ≥1 (OR 2.5, 95% CI: 1.2–5.0, P = 0.01) and NLR ≥3 (OR 3.7, 95% CI: 1.0–3.4, P <0.05) remained independently associated with 28-day mortality. On multivariate binary logistic regression analysis of factors associated with 6-month mortality, PESI score ≥100 (OR 6.2, 95% CI: 2.3–17.0, p<0.001) and coronary artery calcification score ≥6 (OR 2.3, 95% CI: 1.1–4.8, p = 0.030) remained independently associated with death at 6-months. When patients who had an underlying cancer diagnosis were excluded from the analysis only GPS≥1 remained independently associated with 6-month mortality (OR 5.0, 95% CI 1.2–22.0, p<0.05).

**Conclusion:**

PESI score >100, poGPS≥1, NLR ≥3 and CAC score ≥6 were associated with 28-day and 6-month mortality. PESI score ≥100, poGPS≥1 and NLR ≥3 remained independently associated with 28-day mortality. PESI score ≥100 and CAC score ≥6 remained independently associated with 6-month mortality. When patients with underlying cancer were excluded from the analysis, GPS≥1 remained independently associated with 6-month mortality. The role of the systemic inflammatory response (SIR) in determining treatment and prognosis requires further study. Routine reporting of CAC scores in CTPA scans for acute PE may have a role in aiding clinical decision-making regarding treatment and prognosis.

## Introduction

The spectrum of presentation for patients with acute pulmonary embolism (PE) ranges from incidentally discovered asymptomatic emboli to massive life-threatening embolism [1]. The wide range of baseline physiology, laboratory profiles, radiological findings and clinical outcomes make the management of acute PE difficult to standardise as the pathophysiologic mechanisms differentiating low-risk and high-risk PE are poorly understood. Therefore, currently available risk-stratification scores may be insufficient to guide management for these patients. For example, some acute PE treatment decision pathways promote a reliance on sustained hypotension, whilst others include the finding of right heart strain on Computerised Tomography Pulmonary Angiography (CTPA) [2, 3]. Similarly, currently available treatment guidelines fail to provide a risk/benefit context (e.g. age stratification, underlying cancer diagnosis) to aid clinicians in deciding the most appropriate treatment strategy. Furthermore, information that is easily measured on CTPA, such as coronary artery calcification (CAC), has not routinely been reported or included in decision making algorithms for patients with acute pulmonary embolus. This is despite some existing evidence to suggest this may be associated with mortality independent of PE severity [4].

To the best of our knowledge, no scoring system for prediction of mortality in patients with acute PE has integrated objective measurements of clinical, laboratory and CTPA findings. The aim of the present study was to utilise all information commonly available to a hospital clinician in order to investigate whether significant associations exist between CTPA findings, haemodynamic parameters and laboratory data, and to place this data in the context of 28-day and 6-month mortality in patents diagnosed with an acute PE.

## Patients and methods

Radiological reports of all patients who underwent a CTPA scan for the investigation of PE in Greater Glasgow and Clyde, Scotland, between 1^st^ January and 30^th^ June 2016 were examined. Caldecott guardian approval was obtained in line with NHS policy.

Following removal of duplicates, a retrospective case-note review of patients who were identified to have had a CTPA confirmed diagnosis of PE was undertaken. Patients were excluded if they received a diagnosis of pneumonia or other intra-thoracic infectious process. Clinical, laboratory and radiological findings were recorded and compared to 28-day and 6-month mortality. Clinical data comprising: presenting complaint, physiological parameters (pulse, blood pressure, temperature, respiratory rate and oxygen saturations), medications and past medical history were recorded from the admission. Laboratory data, which comprised of full blood count (FBC), D-dimer and biochemical assays (including highly sensitive troponin (hsTnI)) were also documented along with biochemical markers of high- and low-grade inflammation (serum C-reactive protein (CRP) and albumin concentrations). Biochemical thresholds that reflect low-grade inflammation, serum CRP >10mg/L and low serum albumin concentrations <35 g/L, were combined in the Glasgow Prognostic Score (GPS) (Table 1) [5]. Biochemical thresholds of high-grade inflammation, serum CRP >150mg/L and low serum albumin concentrations <25 g/L, were combined in the peri-operative Glasgow Prognostic Score (po-GPS) (Table 2) [6]. Neutrophil Lymphocyte Ratio (NLR) was also calculated (Table 3) [7]. NLR is calculated by dividing neutrophil count by lymphocyte count in a full blood count (FBC) sample result [8]. NLR score ≥3 and NLR ≥5 are indicative of moderately severe and severe inflammation respectively [9, 10].

**Table 1:** Glasgow Prognostic Score (GPS)*.

Glasgow Prognostic Score (oGPS)	Score
**C-reactive protein**	
≤ 10mg/L	**0**
> 10mg/L	**1**
**Albumin**	
≥35 g/L	**0**
<35 g/L	**1**

*note measure of SIRS severity from GPS score, 0 = nil, 1 = mild, 2 = moderate.

**Table 2:** Combined biochemical markers of high-grade inflammation in the peri-operative Glasgow Prognostic Score (poGPS).

peri-operative Glasgow Prognostic Score (poGPS)	Score	SIRS Severity
**C-reactive protein ≤ 150mg/l and Albumin ≥25 g/l**	0	Mild
**C-reactive protein > 150mg/l and Albumin ≥25 g/l**	1	Moderate
**C-reactive protein ≤ 150mg/l and Albumin <25 g/l**	1	Moderate
**C-reactive protein > 150mg/l and Albumin <25 g/l**	2	Severe

**Table 3:** Calculation of the Neutrophil Lymphocyte Ratio (NLR).

Neutrophil Lymphocyte Ratio (NLR):	Ratio	SIRS Severity
**Neutrophil count: lymphocyte count**	<3	Mild
**Neutrophil count: lymphocyte count**	3–5	Moderate
**Neutrophil count: lymphocyte count**	>5	Severe

Pulmonary Embolism Severity Index (PESI) was also calculated [11] (Table 4). PESI score was not calculated if one or more components of the PESI score were not available e.g. contemporaneous measurement of a physiological parameter.

**Table 4:** PESI scoring system.

Variable		Score
Age (years)*	(years = x)	+ x
Sex	Male	+10
	Female	—
Temperature <36*°*C/96.8*°*F	Yes	+20
	No	—
Systolic Blood Pressure <100mmHg	Yes	+30
	No	—
Heart Rate >110bpm	Yes	+20
	No	—
Respiratory Rate >30bpm	Yes	+20
	No	—
Oxygen Saturation <90%	Yes	+20
	No	—
History of Chronic Pulmonary Disease	Yes	+10
	No	—
History of Heart Failure	Yes	+10
	No	—
Altered Mental Status	Yes	+60
	No	—
Malignancy	Yes	+30
	No	—

All positive CTPA scans were re-reported by a specialist-registered radiologist who was blinded to the physiological data and clinical outcome. Scans were re-scored for right heart strain, obstruction index and coronary artery calcification. Radiological parameters measured included right ventricular: left ventricular ratio (transverse diameter ≥ 1.5cm, and 4-chamber desktop reformat), superior vena cava (SVC) area (mm^2^), pulmonary artery (PA) trunk diameter (mm), PA trunk area (mm^2^), azygous vein diameter (mm), inferior vena cava (IVC) reflux, and bowing of interventricular septum. Clot burden/obstruction index was assessed using Qanadli score [12]. Coronary artery calcification (CAC) score was assessed using Weston scoring system [13]. Inter-observer variation was controlled by a second re-reporting of a random selection of scans by another specialist registered radiologist who was also blinded to the previous radiological scores, physiological data and clinical outcomes.

CAC scores reported in the present study were performed retrospectively as CAC scoring was not routinely reported contemporaneously at the time of patient inclusion in the present sample. Therefore, CAC scores did not influence subsequent clinical management (coronary artery angiography, stenting or prescription of secondary prevention medication) for patients in the current study sample.

Treatments were categorised into: no treatment administered; low molecular weight heparin (LMWH) alone; thrombolysis; interventional radiology (embolectomy); direct oral anticoagulation (DOAC) (apixaban or rivaroxaban), and warfarin (with LMWH pre-treatment).

### Statistical analysis

Data was analysed in relation to 28-day and 6-month mortality using Chi-squared and Mann-Whitney U test. Binary logistic regression analysis was performed for variables that achieved p<0.1 on univariate analysis. Calculation of non-parametric bivariate correlations were conducted using Spearman’s rank correlation to calculate the correlation coefficient Rho (r_s_). When taken at p<0.05, r_s_ values 0.00–0.100; 0.100–0.39; 0.40–0.69; 0.70–0.89 and 0.90–1.0 indicate negligible, weak, moderately strong, strong and very strong to perfect monotonic relationships respectively between variables [14]. All data was analysed using SPSS version 27.

## Results

A total of 245 positive PE reports were identified from a total of 2,129 CTPA reports. The majority of patients were female (52%) and older than 65 years old (63%). Of note, 20% of patients had a diagnosis of active cancer. Of 245 patients in the sample, 20 (8%) patients had died at 28-days and 43 (18%) had died at 6-months following CTPA. Of the 197 non-cancer related presentations (102 women (52%)), the overall 28-day and 6-month mortality were 3% and 8% respectively.

PESI score was not calculated for 6 patients (2.5%) as contemporaneous measurements of one or more physiological components of the PESI score were not available. In the present study, PESI score ≥100 (n = 239) was associated with CTPA calcium score ≥6 and IVC reflux (both p<0.001), however PESI score ≥100 was not associated with bowing of the interventricular septum or right-to-left ventricular ratio ≥ 1 **(**RV: LV ≥1).

Chest pain (40%), shortness of breath (43%) and syncope (5%) were the most common presenting complaints. No association between presenting complaint and final outcome was found, however chest pain and shortness of breath were associated with a PESI score ≥100 (p<0.001) and a NLR ratio ≥3 (p<0.05).

There was no evidence of an association between D-dimer categories and mortality (Tables 4–7), however D-dimer concentrations were associated with obstruction index ≥20 (p<0.001).

**Table 5:** The relationship between baseline characteristics and all cause 28-day mortality in patients with acute pulmonary embolus (n = 245).

	Alive (n = 225)	Dead (n = 20)	*P*-value^1^	O.R.	Limits	*P*-value^2^
Sex (male/female)	108/117	9/11	0.797			
Age (years)	69 (55–77)	73 (62–81)	0.156			
Age (< 65/ ≥ 65 years)	85/140	5/15	0.257			
**Clinical**						
Pulse (bpm)	95 (80–110)	97 (84–126)	0.391			
Pulse (</ ≥ 110 bpm)	166/55	14/6	0.615			
Systolic BP (mmHg)	120 (105–139)	115 (92–126)	0.069			
Systolic BP (≥/< 110 mmHg)	151/69	12/7	0.623			
Diastolic BP (mmHg)	76 (66–88)	51 (44–78)	0.263			
Diastolic BP (≥/< 65 mmHg)	169/51	12/7	0.184			
Mean Arterial pressure (mmHg)	94 (84–107)	88 (74–97)	0.098			
Mean Arterial pressure (≥/<80mmHg)	181/39	13/6	0.139			
Respiratory rate (min^-1^)	18 (16–22)	19 (17–22)	0.765			
Respiratory rate (</ ≥ 30 min^-1^)	209/14	18/2	0.512			
SaO_2_ (%)	96 (93–98)	93 (89–96)	0.008			
SaO_2_ (≥ 94% /< 94%)	162/63	9/11	0.012			
Temperature (°C)	36.5 (36.1–37)	36.3 (35.7–36.8)	0.199			
Temperature (≥/< 36°C)	185/36	13/7	0.037			
Consciousness (A/V/P/U)	217/6/1/1	18/0/0/2	0.005			
Active cancer (yes/no)	34/191	14/6	<0.001			
Heart failure (yes/no)	22/203	4/16	0.156			
Chronic lung disease (yes/no)	55/168	8/12	0.135			
PESI	98 (78–126)	132 (118–153)	<0.001			
PESI (<100/ ≥ 100)	109/111	2/17	<0.001	5.2	1.1–24.2	0.036
Treatment 0–5	2/104/4/1/110/4	1/16/0/1/2/0	0.001			
**Laboratory**						
CRP (mg/L)	34 (15–82)	84 (66–237)	0.001			
CRP (<10/10-80/ >80 - <150 / ≥150 mg/L)	27/132/30/25	1/9/4/6	0.011			
Albumin (g/L)	33 (28–37)	27 (21–29)	<0.001			
Albumin (≥35/ 25 - <35/ <25 g/L)	90/116/16	0/11/9	<0.001			
GPS (0/1/2)	18/73/119	0/1/19	0.002			
poGPS (0/1/2)	176/30/6	9/6/5	<0.001	2.5	1.2–5.0	0.010
Haemoglobin (g/L)	131 (114–144)	116 (105–127)	0.027			
Neutrophils (x10^9^/L)	6.4 (5.1–9.0)	11.3 (7.6–14.1)	<0.001			
Neutrophils (</≥ 7.5 x 10^9^ /L)	136/87	5/15	0.002			
Lymphocytes (1.1–5.0 x 10^9^ /L)	1.5 (1.0–2.1)	0.95 (0.7–1.6)	0.014			
Lymphocytes (≥/<1.1 x 10^9^ /L)	168/56	8/12	0.001			
NLR	4.6 (2.8–7.2)	9.2 (7.0–19.8)	<0.001			
NLR (<3 / ≥3 - <5 / ≥5)	68/52/103	0/2/18	<0.001	3.7	1.0–3.4	0.043
NLR (</≥4)	121/103	2/18	<0.001			
D-dimer (ng/dL)	1417 (667–3187)	1921 (1167–2937)	0.399			
D-dimer < 230 / ≥ 230 ng/dL	3/190	0/8	0.723			
High sensitivity Troponin (ng/L)*	20.5 (4–115)	10.5 (5.0–50)	0.561			
hsTnI (≤5/ 5–16 or 34 / >16 or 34 ng/dL)*	43/33/58	1/4/1	0.753			
**Radiological**						
Obstruction count	11 (4–19)	7.5 (2.0–12.0)	0.035			
Obstruction index	28 (10–46)	18.7 (5.0–30)	0.042			
Qanadli Score < / ≥ 20	95/128	10/10	0.523			
4 chamber RV:LV</≥1.0	125/100	11/9	0.962			
Bowing of Inter-ventricular septum (no bowing /flat/bowed)	150/41/38	16/3/1	0.509			
SVC area (mm^2^)	276 (205–347)	277 (228–295)	0.456			
PA trunk area (mm^2^)	643 (548–808)	620 (512–759)	0.487			
Azygous vein diameter	7.0 (6.0–8.0)	6.0 (5.0–7.0)	0.290			
IVC reflux (0–3)	135/42/23/25	12/4/2/2	0.919			
Emphysema score (0–4)	155/34/20/15/1	11/2/1/6/0	0.017			
Coronary calcification	3.0 (0–7.0)	7.5 (1.3–9.0)	0.092			
Coronary calcification (</≥6)	147/77	8/12	0.023	-	-	0.475

*MAP: mean arterial pressure

** 0 = no treatment, 1 = Low molecular weight heparin, 2 = thrombolysis, 3 embolectomy, 4 apixaban/rivaroxaban 5. warfarin.

**Table 6:** The relationship between baseline characteristics and all-cause 6-month mortality for patients with acute pulmonary embolus (n = 245).

	Alive (n = 202)	Dead (n = 43)	*P*-value^1^	O.R.	Limits	*P*-value^2^
Age (< 65/ ≥ 65 years)	82/120	8/35	0.007			
Sex (male/female)	95/107	22/21	0.623			
**Clinical**						
Pulse (< 100 / ≥ 100 bpm)	150/49	30/12	0.594			
Systolic BP (≥/< 110 mmHg)	136/62	27/14	0.723			
Diastolic BP (≥/< 65 mmHg)	157/41	24/17	0.005			
MAP* (≥/< 80mmHg)	166/32	28/13	0.021			
Respiratory rate (</≥ 30 min^-1^)	188/13	39/3	0.873			
SaO_2_ (≥ 94% /< 94%)	146/56	25/18	0.067			
Temperature (≥/< 36°C)	168/31	30/12	0.046			
Consciousness (A/V/P/U)	196/4/1/1	39/2/0/2	0.028			
Active cancer (yes/no)	20/182	28/15	<0.001			
Heart failure (yes/no)	18/184	8/35	0.061			
Chronic lung disease (yes/no)	44/156	19/24	0.003			
PESI (<100/ ≥ 100)	105/93	6/35	<0.001	6.2	2.3–17.0	<0.001
Treatment** 0–5	2/89/4/1/102/4	1/31/0/1/10/0	<0.001			
**Laboratory**						
CRP (ng/L)	42 (17–89)	67 (20–122)	0.023			
CRP (<10/10-80/ >80 - <150 / ≥150 mg/L)	25/116/27/24	3/25/7/7	0.243			
Albumin (g/L)	33 (28–36)	27 (23–30)	<0.001			
Albumin (≥35 / 25 - <35/ <25 g/L)	89/96/14	1/31/11	<0.001			
GPS (0/1/2)	18/71/99	0/3/39	<0.001			
poGPS (0/1/2)	157/27/6	28/9/5	0.006	-	-	0.141
NLR (<3 / ≥3 - <5 / ≥5)	62/47/91	6/7/30	0.004	-	-	0.185
Hb (g/L)	129 (113–144)	115 (106–133)	0.003			
D-dimer (ng/dL)	1418 (660–3178)	1421 (932–3159)	0.450			
D-dimer < 230 / ≥ 230 ng/dL	3/169	0/29	0.475			
hsTnI (≤5/ 5–16 or 34 / >16 or 34 ng/dL)*	40/26/55	4/11/4	0.556			
**Radiological**						
Obstruction count	12 (4–19)	7 (2–14)	0.004			
Obstruction index	30.0 (10.0–48.0)	17.5 (5.0–35.0)	0.006			
Qanadli Score < / ≥ 20	82/119	23/19	0.097			
4 chamber RV:LV</≥1.0	110/92	26/17	0.472			
Bowing of Inter-ventricular septum (no bowing /flat/bowed)	134/35/33	32/6/5	0.427			
SVC area (mm^2^)	277 (208–351)	273 (213–319)	0.420			
PA trunk area (mm^2^)	636 (540–806)	667 (576–806)	0.731			
Azygous vein diameter	7.0 (6.0–8.0)	6.0 (5.0–7.0)	0.247			
IVC reflux (0–3)	125/35/21/21	22/11/4/6	0.335			
Emphysema score (0–4)	144/27/16/14/1	22/9/5/7/0	0.014			
Coronary calcification	2 (0–7)	6 (2–9)	0.001			
Coronary calcification (</≥6) (all cases)	94/32	16/18	<0.001	2.3	1.1–4.8	0.030

*MAP: mean arterial pressure

** 0 = no treatment, 1 = Low molecular weight heparin, 2 = thrombolysis, 3 embolectomy, 4 apixaban/rivaroxaban, 5. warfarin.

**Table 7:** The relationship between baseline characteristics and all cause 28-day mortality in non-cancer patients with acute pulmonary embolus (n = 197).

	Alive (n = 191)	Dead (n = 6)	*P*-value^1^
Sex (male/female)	92/99	3/3	0.930
Age (years)	68 (54–77)	71 (58–85)	0.485
Age (< 65/ ≥ 65 years)	75/116	2/4	0.770
**Clinical**			
Pulse (bpm)	94 (80–108)	85 (76–102)	0.402
Pulse (</ ≥ 110 bpm)	145/44	6/0	0.180
Systolic BP (mmHg)	120 (105–139)	91 (84–121)	0.020
Systolic BP (≥/< 110 mmHg)	129/59	2/4	0.070
Diastolic BP (mmHg)	67 (55–75)	51 (44–60)	0.032
Diastolic BP (≥/< 65 mmHg)	104/85	1/5	0.064
Mean Arterial pressure (mmHg)	84 (74–96)	54 (68–80)	0.015
Mean Arterial pressure (≥/<80mmHg)	113/75	1/5	0.034
Respiratory rate (min^-1^)	18 (16–22)	17 (15–23)	0.396
Respiratory rate (</ ≥ 30 min^-1^)	179/12	5/1	0.314
SaO_2_ (%)	96 (93–98)	91 (85–97)	0.138
SaO_2_ (≥ 94% /< 94%)	140/51	3/3	0.209
Temperature (°C)	36.5 (36.1–37)	36.2 (35.4–36.9)	0.227
Temperature (≥/< 36°C)	161/28	4/2	0.217
Consciousness (A/V/P/U)	186/3/1/1	5/0//0/1	0.002
Heart failure (yes/no)	16/175	3/3	0.001
Chronic lung disease (yes/no)	146/43	3/3	0.123
PESI	96 (75–116)	127 (99–171)	0.027
PESI (<100/ ≥ 100)	107/81	1/5	0.051
Treatment 0–5	2/76/3/1/105/4	1/4/0/0/1/0	0.025
**Laboratory**			
CRP (mg/L)	29 (14–83)	133 (51–278)	0.050
CRP (<10/10-80/ >80 - <150 / ≥150 mg/L)	25/109/24/22	1/1/1/3	0.040
Albumin (g/L)	34 (29–37)	28 (26–29)	0.007
Albumin (≥35/ 25 - <35/ <25 g/L)	86/88/14	0/5/1	0.034
GPS (0/1/2)	17/69/91	0/1/5	0.129
poGPS (0/1/2)	147/25/6	3/2/1	0.027
Haemoglobin (g/L)	132 (116–145)	118 (105–152)	0.504
Neutrophils (x10^9^/L)	6.4 (5.0–8.9)	8.9 (8.0–11.6)	0.024
Neutrophils (</≥ 7.5 x 10^9^ /L)	117/72	1/5	0.026
Lymphocytes (1.1–5.0 x 10^9^ /L)	1.5 (1.1–2.1)	0.9 (0.8–2.0)	0.166
Lymphocytes (≥/<1.1 x 10^9^ /L)	150/40	2/4	0.009
NLR	4.3 (2.5–6.9)	8.9 (5.2–13.0)	0.027
NLR (<3 / ≥3 - <5 / ≥5)	61/47/81	0/1/5	0.042
D-dimer (ng/dL)	1418 (667–3187)	1108 (918)^	0.745
D-dimer < 230 / ≥ 230 ng/dL	2/165	0/3	0.849
High sensitivity Troponin (ng/L)*	23 (4–126)	6 (2)^	0.237

*MAP: mean arterial pressure

** 0 = no treatment, 1 = Low molecular weight heparin, 2 = thrombolysis, 3 embolectomy, 4 apixaban/rivaroxaban, 5. warfarin.

Sex specific thresholds of high sensitivity troponin indicating myocardial injury (hsTnI >16 or >34ng/L for female and male respectively) [15, 16] were associated with bowing of the inter-ventricular septum on CTPA (p = 0.001). High sensitivity troponin (hsTnI) values >5ng/L correlated with PESI score (r_s_ = 0.407; p<0.001; n = 138), D-dimer concentrations (r_s_ = 0.452; p<0.001; n = 129), higher obstruction index (r_s_ = 0.411; p<0.001; n = 140), bowing of the interventricular septum (r_s_ = 283; p<0.001; n = 140) and coronary artery calcification score (r_s_ = 269; p<0.001; n = 140).

On non-parametric univariate analysis of continuous data (Mann Whitney U test), initial SaO2 (p<0.01), PESI score (p<0.001), NLR (p<0.001), CRP (p = 0.001) and albumin (p<0.001) were associated with higher 28-day mortality (Table 5). On Chi-squared analysis, PESI score ≥100 (p<0.001 and NLR ≥3 (p<0.001) were associated with higher 28-day mortality. Biochemical thresholds of high-grade inflammation, serum CRP >150mg/L and low serum albumin concentrations <25 g/L, combined in the poGPS (p<0.001) were associated with higher 28-day mortality (Table 5). Of the radiological measurements, emphysema score (p = 0.017) and coronary artery calcification score ≥6 (p = 0.023) were associated with higher 28-day mortality on Mann Whitney U test and Chi-squared test respectively (Table 5). On multivariate binary logistic regression analysis, PESI score ≥100 (OR 5.2, 95% CI: 1.1, 24.2, P <0.05), poGPS ≥1 (OR 2.5, 95% CI: 1.2–5.0, P = 0.01) and NLR ≥3 (OR 3.7, 95% CI: 1.0–3.4, P <0.05) remained independently associated with 28-day mortality (Table 5).

On univariate analysis, age ≥65 years (p = 0.007), diastolic BP <65 mmHg (p = 0.005), mean arterial pressure (MAP) <80 mmHg (p = 0.021), PESI score ≥100 (<0.001), albumin <35 g/L (p<0.001), poGPS>1 (p = 0.006), NLR ≥3 (p = 0.04) and CTPA coronary artery calcification score ≥6 (p<0.001) were associated with higher 6-month mortality (Table 6). On multivariate binary logistic regression analysis of factors associated with 6-month mortality, PESI score ≥100 (OR 6.2, 95% CI: 2.3–17.0, p<0.001) and coronary artery calcification score ≥6 (OR 2.3, 95% CI: 1.1–4.8, p = 0.030) remained independently associated with death at 6-months (Table 6); however, when multivariate analysis was conducted with active cancer status (and eligible components of PESI score) replacing PESI score, a stronger independent association was found between active cancer and 6-month mortality (OR 14.5, 95% CI 6.3–33.0, p<0.001).

### Cancer and mortality

48 (20%) of the total patient sample (n = 245) had a concurrent diagnosis of cancer; 14 (29%) and 28 (58%) of these patients had died at 28-days and 6-months respectively post PE-diagnosis. The Glasgow Prognostic Score (GPS) (CRP≤10 = 0, >10 = 1; albumin ≥35 = 0, <35 = 1) is a validated mortality prediction score used in cancer patients (Table 1) [17]. When GPS was applied to the cancer group, 6-month mortality rates increased with increasing GPS (0 = 0%, 1 = 2%, 2 = 57%).

### Non-cancer mortality

When patients who had an underlying cancer diagnosis were excluded from the analysis, 6 (3%) and 15 (8%) patients died at 28-days and 6-months respectively. MAP<80mmHg (p<0.01), past medical history of heart failure (p<0.05), PESI score (p<0.05), poGPS ≥1 (p<0.05), NLR≥3 (p<0.05) remained significantly associated with increased 28-day mortality (p<0.05) (Table 7).

When patients who had an underlying cancer diagnosis were excluded from the analysis, diastolic BP <65mmHg, MAP<80mmHg (both p<0.01), past medical history of heart failure (p<0.05), past medical history of chronic lung disease (p<0.05), PESI score (p<0.05), GPS≥1 (p<0.05) and NLR≥3 (p<0.05) remained significantly associated with 6-month mortality. On binary logistic regression analysis, only GPS≥1 remained independently associated with 6-month mortality (OR 5.0, 95% CI 1.2–22.0, p<0.05) (Table 8).

**Table 8:** The relationship between baseline characteristics and all cause 6-month mortality in non-cancer patients with acute pulmonary embolus (n = 197).

	Alive (n = 182)	Dead (n = 15)	*P*-value^1^	O.R.	Limits	*P*-value^2^
Age (years)	68 (54–76)	75 (67–85)	0.034			
Age (< 65/ ≥ 65 years)	74/108	3/12	0.116			
Sex (male/female)	89/93	6/9	0.508			
**Clinical**						
Pulse (< 100 / ≥ 100 bpm)	139/41	12/3	0.805			
Systolic BP (≥/< 110 mmHg)	124/55	7/8	0.073			
Diastolic BP (≥/< 65 mmHg)	102/78	3/12	0.006			
MAP* (≥/< 80mmHg)	110/69	4/11	0.009			
Respiratory rate (</≥ 30 min^-1^)	171/11	13/2	0.276			
SaO_2_ (≥ 94% /< 94%)	133/49	10/5	0.594			
Temperature (≥/< 36°C)	152/28	13/2	0.819			
Consciousness (A/V/P/U)	177/3/1/1	14/0/0/1	0.100			
Heart failure (yes/no)	15/167	4/11	0.020			
Chronic lung disease (yes/no)	39/141	7/8	0.029			
PESI	92 (73–116)	115 (96–138)	0.021			
PESI (<100/ ≥ 100)	103/76	5/10	0.071	-	-	0.224
Treatment** 0–5	2/72/3/1/100/4	1/8/0/0/6/0	0.130			
**Laboratory**						
CRP (<10/10-80/ >80 - <150 / ≥150 mg/L)	24/104/20/24	2/6/3/3	0.318			
Albumin (≥35 / 25 - <35/ <25 g/L)	85/81/13	1/2/2	0.005			
GPS (0/1/2)	17/68/84	0/2/12	0.012	5.0	1.2–22.0	0.030
poGPS (0/1/2)	140/24/6	10/3/1	0.296			
Neutrophils (x10^9^/L)	6.5 (5.0–8.9)	8.3 (5.7–10.1)	0.225			
Neutrophils (</≥ 7.5 x 10^9^ /L)	111/69	7/8	0.255			
Lymphocytes (1.1–5.0 x 10^9^ /L)	1.5 (1.1–2.1)	1.1 (0.9–1.9)	0.046			
Lymphocytes (≥/<1.1 x 10^9^ /L)	144/37	8/7	0.020			
NLR	4.2 (2.6–6.9)	7.3 (3.6–10.0)	0.039			
NLR (<3 / ≥3 - <5 / ≥5)	59/44/77	2/4/9	0.113	-	-	
D-dimer (ng/dL)	1418 (665–3164)	1421 (932–3159)	0.507			
D-dimer < 230 / ≥ 230 ng/dL	2/156	0/12	0.696			
hsTnI (≤5/ 5–16 or 34 / >16 or 34 ng/dL)*	34/26/48	3/4/3	0.648			
**Radiological**						
Obstruction count	13 (5–19)	6 (4–14)	0.058			
Obstruction index	33.5 (12.0–48.0)	15 (10.0–35.0)	0.069			
Qanadli Score < / ≥ 20	68/113	8/6	0.149			
4 chamber RV:LV</≥1.0	98/84	9/6	0.646			
Bowing of Inter-ventricular septum (no bowing /flat/bowed)	121/32/29	10/1/4	0.460			
SVC area (mm^2^)	277 (206–352)	282 (228–312)	0.865			
PA trunk area (mm^2^)	631 (535–802)	692 (584–828)	0.318			
Azygous vein diameter	7.0 (6.0–8.0)	7.0 (5.0–7.0)	0.472			
IVC reflux (0–3)	113/31/20/18	9/3/1/2	0.865			
Emphysema score (0–4)	128/27/15/11/1	8/5/1/1/0	0.542			
Coronary calcification	2 (0–7)	6 (2–11)	0.031			
Coronary calcification (< / ≥ 6)	126/55	7/8	0.068	-	-	0.059

*MAP: mean arterial pressure

** 0 = no treatment, 1 = Low molecular weight heparin, 2 = thrombolysis, 3 embolectomy, 4 apixaban/rivaroxaban, 5. warfarin.

### Treatment

Overall, only 4 patients (1.6%) received thrombolysis and 2 (0.8%) underwent interventional radiology treatment (embolectomy). Almost half of the patients (49%) in the present sample received treatment with low molecular weight heparin (LMWH) (dalteparin) and 46% received Direct Oral Anticoagulation (DOAC) medication (apixaban/rivaroxaban). When treatment with LMWH alone was compared against all other treatments using Chi-squared analysis, treatment with LMWH alone was associated with increased 28-day (p = 0.004) and 6-month mortality (p = 0.001). However, when patients with an underlying diagnosis of cancer were removed from the analysis, no association was found between treatment category and 28-day (p = 0.188) or 6-month mortality (p = 0.298).

Only 9 (10%) of the 89 patients who had CAC score ≥6 in the present study underwent Coronary Artery Angiography during the 6 months following diagnosis of acute PE.

## Discussion

The percentage of positive scans (11.5%) in this study sample is consistent with that reported in other studies [18] and mortality figures are consistent with the prospective validation of the PESI score reported by Donzé *et al*. [11].

In the present study, patients with a PESI score ≥100 were more than 5 times more likely to die before 28-days. PESI score was also associated with CTPA coronary artery calcium score ≥6 and 6-month mortality.

Right ventricular ischemia has been proposed as a mechanism for RV dysfunction in acute pulmonary embolism [19, 20]. It is therefore of interest that coronary artery calcification score ≥6 was associated with increased mortality at 6-months in patients with acute PE in the present study. These results may reflect a reduced capacity of under-perfused myocardium to respond to increased right ventricular afterload following PE.

**Fig 1:**
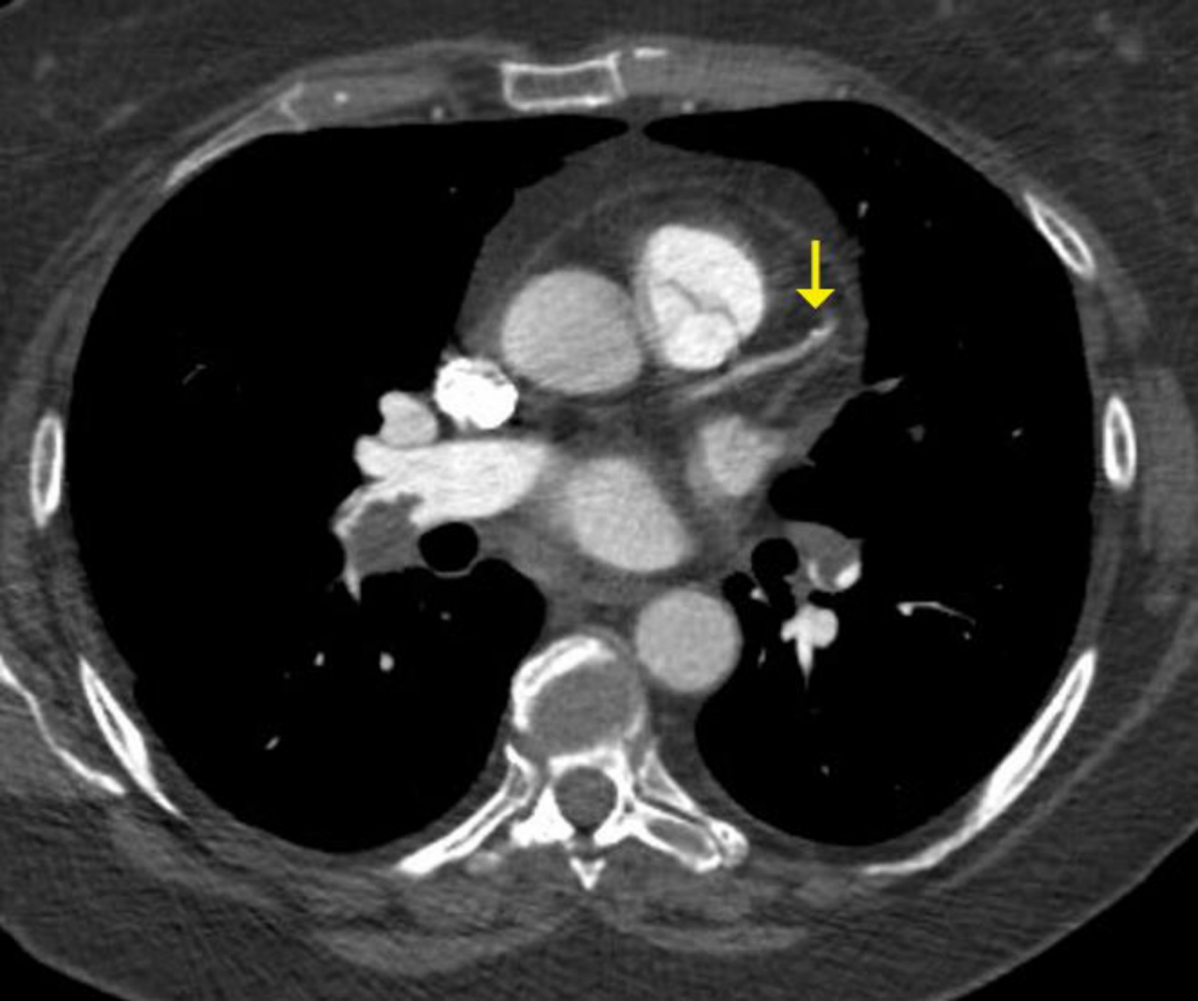
Low coronary artery calcium score–single tiny calcified plaque distal left anterior descending coronary artery (arrow).

**Fig 2:**
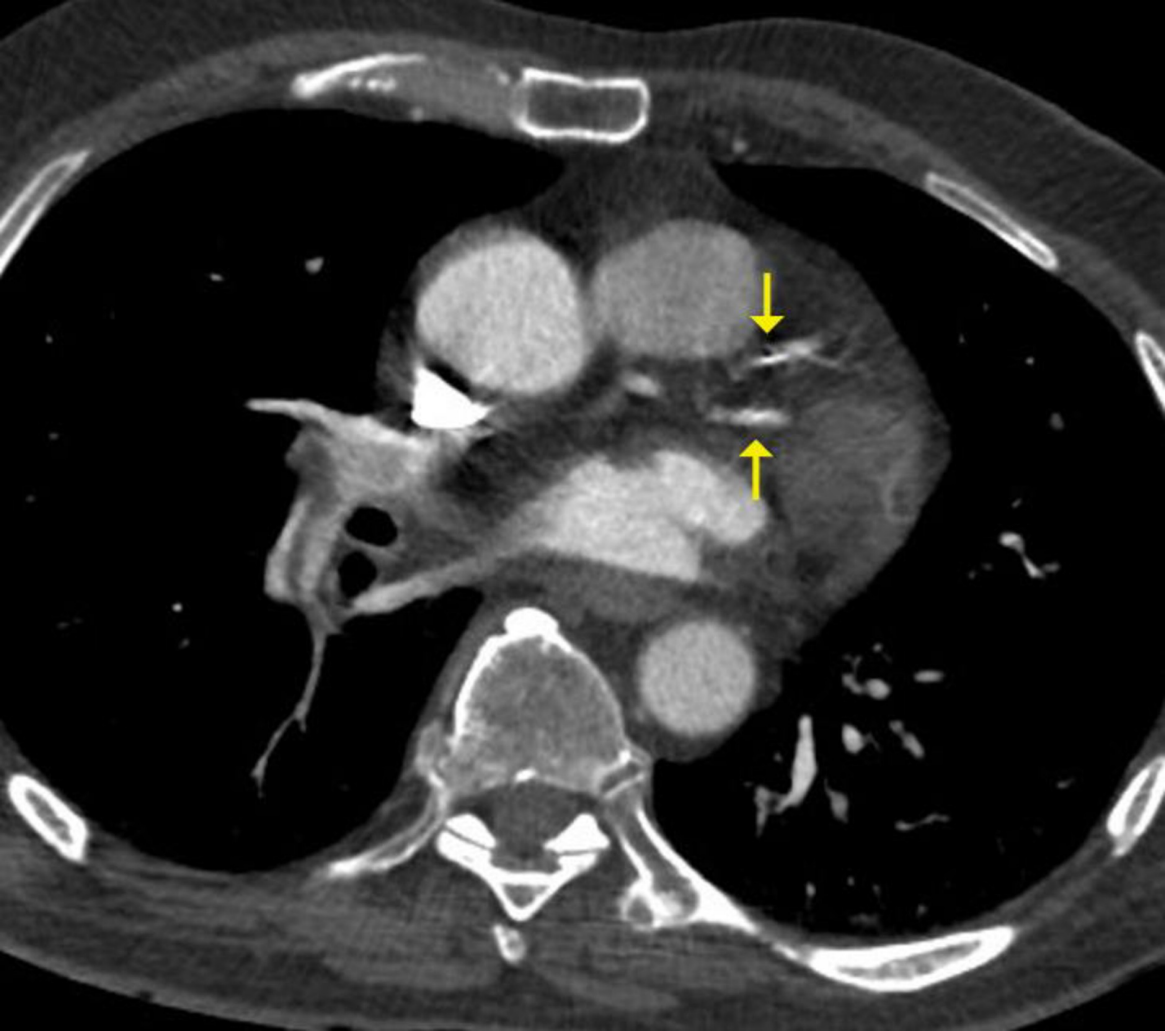
Moderate coronary artery calcium score–calcified plaques in both left anterior descending and intermediate left coronary artery branches (arrows).

**Fig 3:**
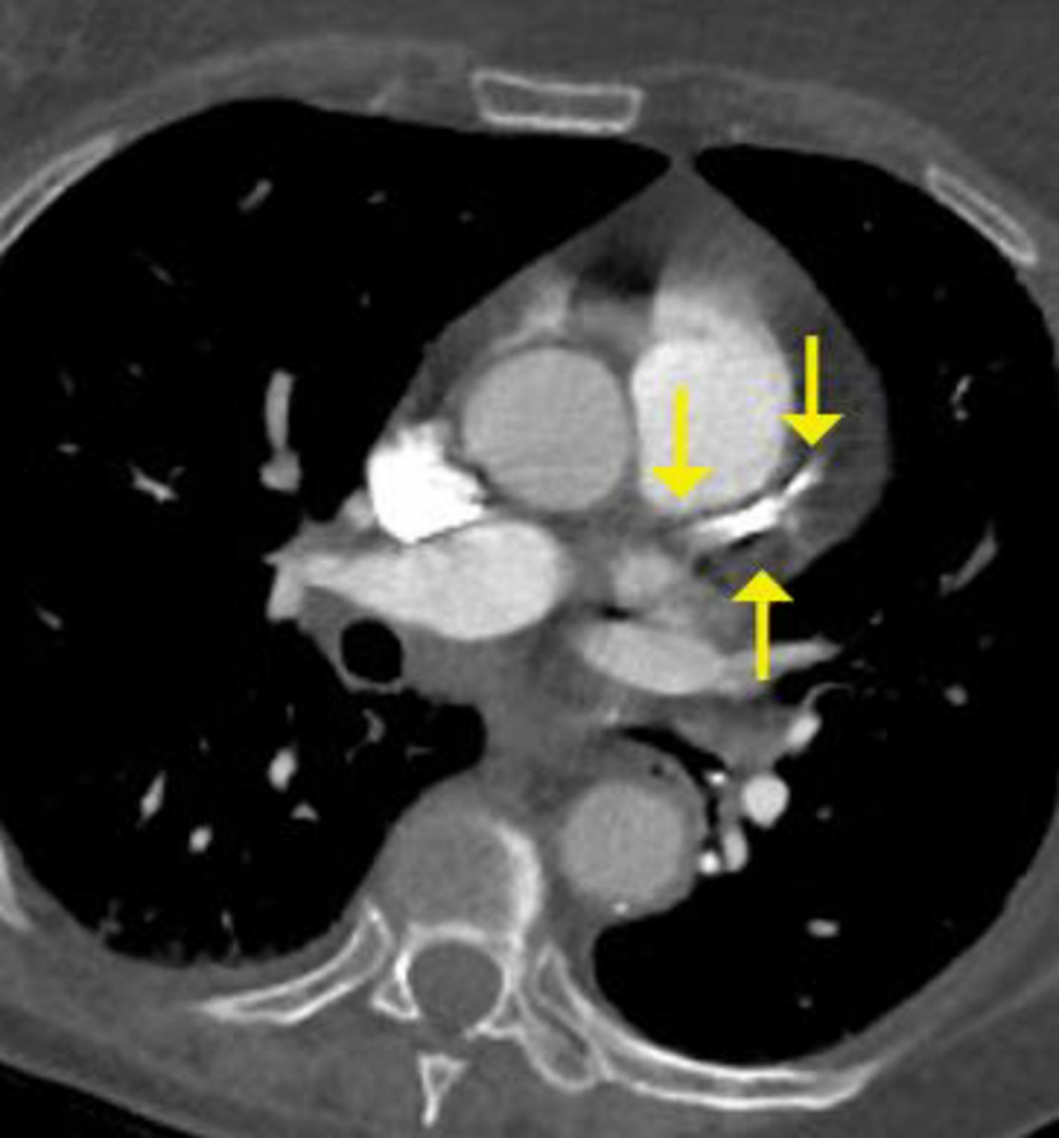
High coronary artery calcium score–extensive and dense calcified plaque in left anterior descending coronary artery (arrows).

Coronary artery calcification is a frequent incidental finding on CTPA, however radiologists infrequently report CTPA coronary artery calcification score when reporting scans performed for the investigation of suspected PE [4, 21]. Williams *et al*. have recently reported that the presence of coronary artery calcification identified on CTPA is a more important predictor of all-cause 3-year mortality than the severity of PE [21]. In their cohort, patients with severe calcification were reported to be almost five-times more likely to die at 3-years when compared to patients without coronary artery calcification [21]. To the best of our knowledge, the results of the present study are the first to report an association between coronary artery calcification and 28-day and 6-month mortality in patients with acute PE. Moreover, coronary artery calcification remained independently associated with 6-month mortality in patients with acute PE. Patients with CAC≥6 were more than two times more likely to die at six months than patients with CAC<6. Therefore, the results of the present study may suggest that routine reporting of coronary artery calcification (CAC) score should be considered for patients undergoing CTPA investigation of suspected PE (Figs 1–3).

The correlation of high sensitivity troponin (hsTnI) concentrations >5ng/L with PESI score ≥100, D-dimer concentrations >230 ng/L, higher obstruction index and bowing of the inter-ventricular septum, indicate that even relatively modest elevation of hsTnI concentrations (into the gender specific intermediate risk intervals [16]) may be of clinical significance in the context of PE.

The results of the present study also suggest that evaluation of the severity of the systemic inflammatory response (SIR) involved in acute pulmonary embolus may have a role in assessing prognosis and guiding clinical decision making [7]. There are a variety of methods that may be used to calculate systemic inflammation; however there is no robust evidence to suggest which score is superior in the context of acute PE. To the best of our knowledge, the results of the present study are the first to highlight the role of biochemical markers of low- and high-grade inflammation (serum C-reactive protein (CRP) and albumin concentrations). Biochemical thresholds that reflect low-grade inflammation, serum CRP >10mg/L and low serum albumin concentrations <35 g/L, combined in the Glasgow Prognostic Score (GPS) remained independently prognostic for patients without an underlying diagnosis of cancer at 6-months post diagnosis of acute PE. In this group, patients with GPS≥1 were five times more likely to die at 6-months than patients with GPS = 0 at time of diagnosis of acute PE. Similarly, biochemical thresholds of high-grade inflammation, serum CRP >150mg/L and low serum albumin concentrations <25 g/L, combined in the poGPS, remained independently associated with higher 28-day mortality. In this group, patients with poGPS = 1 and poGPS = 2 were 2.5 and 5 times respectively more likely to die at 28-days than patients with poGPS = 0 at time of diagnosis of acute PE.

Neutrophil to Lymphocyte Ratio (NLR) is also a widely accepted measure of systemic inflammation that has been reported to have prognostic value in several clinical situations including pulmonary embolus [7]. NLR has been reported to be independently associated with short and medium term mortality in patients with PE [7, 8], and the results of the present study indicate that patients with a NLR ≥3 and ≥5 were almost 4 and 7.5 times respectively more likely to die before 28-days.

However, NLR is also a prognostic marker for numerous cancers [10, 22] and this raises the possibility of confounding within scoring systems that include active cancer diagnosis. For example, active cancer is heavily weighted in the PESI score. It is therefore of interest in the present study that NLR remained significantly associated with both 28-day and 6-month mortality when patients who had an underlying cancer were excluded from the analysis.

Similarly, high serum CRP and low serum albumin concentrations are prognostic markers for numerous cancers [23]. However, when patients who had an underlying cancer were excluded from the analysis and CRP and albumin were combined in the measure of high-grade inflammation, poGPS remained significantly associated with higher 28-day mortality. Moreover, when patients who had an underlying cancer were excluded from the analysis, and CRP and albumin were combined in the measure of low-grade inflammation, GPS remained independently associated with higher 6-month mortality.

Overall, these results may indicate that the systemic inflammatory response has a role in pathophysiological mechanisms that contribute to 28-day and 6-month mortality in patients with acute pulmonary embolus. Furthermore, the role of SIR in the context of acute PE is of greater interest when it is considered that clinical indicators that currently influence treatment decisions, such as systolic hypotension, RV:LV ratio≥1.0, or bowing of the inter-ventricular septum, have not been shown to have a significant association with 28-day or 6-month mortality in the present sample.

Right ventricular myocardial inflammatory response has been described in acute PE, however the role of inflammation in the transition from RV adaptation to RV failure remains incompletely understood [24]. Nonetheless, it is understood that multiple pro-inflammatory changes occur in the RV at a cellular level following acute PE (oxidative stress, apoptosis, infiltration of inflammatory cells, fibrosis, and metabolic reprogramming) [24, 25]. The resulting alteration in RV architecture is reported to result in a spectrum of change that ranges from loss of RV contractility to subsequent RV failure that may result in dysregulated coupling between the RV and the pulmonary circulation [24].

The association between biochemical (GPS and poGPS) and haematological (NLR) inflammatory scores and 28-day and 6-month mortality in the present study is consistent with this evidence base and raises the question of the role of potential anti-inflammatory treatment strategies to mitigate against short- and medium-term mortality following acute PE. Further work may be directed towards the integration of scores that quantify the haemopoietic and metabolic responses to the SIR in acute PE.

In the present study, treatment with LMWH alone was also associated with higher mortality, indicating that clinicians may be undertreating PE with thrombolysis and/or embolectomy. However, this association may be confounded by the fact that the recommended treatment for cancer patients with PE is primarily with LMWH (not DOAC or warfarin), hence explaining the higher mortality in the LMWH alone group. Nonetheless, the association between treatment with LMWH alone and higher 28-day and 6-month mortality may also be reflected in the clinical paradox that higher pulmonary artery obstruction index was associated with better survival at 28-days and 6-months in the present sample.

Given the high 28-day and 6-month mortality of patients who had PE in the context of underlying cancer, PE in these circumstances may represent a pre-terminal event. However, this group warrants further investigation regarding treatment strategies, as treatment of selected patients with PE within the more severe cancer group (GPS = 2) may confer a survival benefit and compliment oncological treatments. Prevention of acute PE in patients with an underlying cancer diagnosis by administration of novel oral anticoagulant (NOAC) or LMWH prophylaxis is gaining traction in the literature [26], and the findings of the present study support this rationale.

Following the results of the PIETHO study [27], clinicians may be reluctant to administer thrombolytic therapy to older patients (>65 years old) with or without an underlying diagnosis of active cancer. The PESI score is heavily weighted for both age and active cancer diagnosis. Therefore, while the PESI score is a useful tool for prediction of mortality, its use in guiding clinicians regarding treatment options for acute PE may be limited. In the future, a score to guide clinicians and patients regarding treatment options may therefore include markers of metabolic distress (e.g. plasma lactate concentrations), inflammation (e.g. poGPS, NLR), measures of physiological compromise (e.g. MAP<80 mmHg; SaO2 stratified for FiO2), biomarkers of myocardial injury (high sensitivity troponin and BNP), radiological evidence of right heart strain (bowing of the septum or RV: LV ratio ≥1.0) combined with measures of clot burden (e.g. Qanadli score) and coronary artery calcification score. Further, larger studies are required to establish the independent short- and medium-term prognostic value of these variables.

### Limitations

Plasma lactate concentrations are reported to be of prognostic significance in acute PE [28]. It is a limitation of the present study that plasma lactate concentrations were infrequently measured in this patient group. Similarly, serum brain natriuretic peptide (pro-BNP) has an established role in diagnosis and prognostication for patients with acute PE [29]. Nonetheless, Pro-BNP measurements were unavailable to clinicians at the time of this study. Also of note, ECG (Electrocardiogram) and ECHO (Echocardiogram) information was not included in this data set.

## Conclusion

Combined biochemical markers of high-grade inflammation (poGPS), serum CRP >150mg/L and low serum albumin concentrations <25 g/L, were associated with 28-day and 6-month mortality in patients with acute PE. PESI score ≥100, NLR ≥3 and coronary artery calcification score ≥6 were also associated with 28-day and 6-month mortality in patients with acute PE. poGPS ≥1, PESI score ≥100 and NLR ≥3 remained independently prognostic with 28-day mortality in patients with acute PE. PESI score ≥100 and coronary artery calcification score ≥ 6 remained independently prognostic with 6-month mortality in patients with acute PE. When patients with an underlying diagnosis of cancer were excluded from the analysis, combined biochemical markers of low-grade inflammation (GPS), serum CRP >10mg/L and low serum albumin concentrations <35 g/L remained independently associated with higher 6-month mortality. At present, no risk stratifying score for acute PE prognosis or treatment includes markers of inflammation or coronary artery calcification scores.

PE in the context of underlying cancer may represent a pre-terminal event as patients were almost 15 times more likely to die at 6-months, however this group warrants further exploration regarding the cause of death, as PE treatment of selected patients within the GPS = 2 group may confer a survival benefit. Thromboprophylaxis in this patient group requires further investigation.
